# Neural tube opening and abnormal extraembryonic membrane development in SEC23A deficient mice

**DOI:** 10.1038/srep15471

**Published:** 2015-10-23

**Authors:** Min Zhu, Jiayi Tao, Matthew P. Vasievich, Wei Wei, Guojing Zhu, Rami N. Khoriaty, Bin Zhang

**Affiliations:** 1 Department of Pathology, Karamay Central Hospital, Karamay, Xinjiang, China; 2 Genomic Medicine Institute, Cleveland Clinic Lerner Research Institute, Cleveland, OH 44195; 3 Life Sciences Institute, University of Michigan, Ann Arbor, MI 48109.

## Abstract

COPII (coat protein complex-II) vesicles transport proteins from the endoplasmic reticulum (ER) to the Golgi. Higher eukaryotes have two or more paralogs of most COPII components. Here we characterize mice deficient for SEC23A and studied interactions of *Sec23a* null allele with the previously reported *Sec23b* null allele. SEC23A deficiency leads to mid-embryonic lethality associated with defective development of extraembryonic membranes and neural tube opening in midbrain. Secretion defects of multiple collagen types are observed in different connective tissues, suggesting that collagens are primarily transported in SEC23A-containing vesicles in these cells. Other extracellular matrix proteins, such as fibronectin, are not affected by SEC23A deficiency. Intracellular accumulation of unsecreted proteins leads to strong induction of the unfolded protein response in collagen-producing cells. No collagen secretion defects are observed in SEC23B deficient embryos. We report that E-cadherin is a cargo that accumulates in acini of SEC23B deficient pancreas and salivary glands. Compensatory increase of one paralog is observed in the absence of the second paralog. Haploinsufficiency of the remaining *Sec23* paralog on top of homozygous inactivation of the first paralog leads to earlier lethality of embryos. Our results suggest that mammalian SEC23A and SEC23B transport overlapping yet distinct spectra of cargo *in vivo.*

Proteins destined for the secretory pathway are synthesized in the endoplasmic reticulum (ER) and transported to the Golgi in coat protein complex II (COPII)-coated vesicles, which are typically 60–80 nm in diameter^1^. COPII consists of a set of five core components: SAR1, SEC23, SEC24, SEC13 and SEC31 and is responsible for the initiation of vesicle formation, cargo selection, polymerization and the release of loaded carriers from the ER membranes^2^,^3^. The process of COPII-coated vesicle formation begins with the conversion of SAR1-GDP into SAR1-GTP at ER exit sites, a process catalyzed by the GTPase exchange protein SEC12. The N-terminal tail of SAR1-GTP is inserted into the ER membrane and initiates membrane curvature. The SEC23-SEC24 complex is then recruited to form the inner layer of the COPII coat, followed by binding of the SEC13-SEC31 complex to form the cage-like outer coat. SEC23 is a GTPase activating protein (GAP) that promotes the conversion of SAR1-GTP back to SAR1-GDP, which promotes coat disassembly^4^. SEC23 may also play a role in cargo recognition^5^, and it sequentially binds to the TRAPP tethering complex and Hrr25p to ensure the directionality of ER-Golgi traffic^6^,^7^.

Mammalian cells contain multiple paralogs of most COPII proteins, including two SEC23 paralogs, SEC23A and SEC23B. Two missense mutations in SEC23A cause cranio-lenticulo-sutural dysplasia (CLSD), a disorder primarily characterized by late-closing fontanels, facial dysmorphisms, skeletal defects and sutural cataracts^8^,^9^. These missense mutations are hypomorphic^10^,^11^ and no null mutations in *SEC23A* have been reported. Mutations in *SEC23B* were subsequently identified as the cause of congenital dyserythropoietic anemia type II (CDAII), a disorder characterized by ineffective erythropoiesis associated with multinucleated erythroblasts^12^,^13^. In contrast, mice with SEC23B deficiency exhibit perinatal lethality, with degeneration of multiple professional secretory tissues with normal skeletal development^14^ and entirely normal erythropoiesis^15^.

The extracellular matrix (ECM) provides structural support for organs and tissues^16^. In mammalian tissues, the ECM is most commonly found in connective tissues such as tendon, cartilage, bone or dermis of the skin. Collagen fibrils in the ECM are the major components of the connective tissues in animals and allow these tissues to withstand tensile forces^17^. Pro-collagens are posttranslationally modified and assembled into triple helix in the ER and evidence indicates that the ER exit of collagens requires COPII^18^. However, the length of a pro-collagen helix assembled in the ER (300–400 nm) greatly exceeds the dimension of a typical COPII vesicle (60–80 nm). It is not yet fully understood how this process is orchestrated and controlled, particularly during embryonic development.

We now report characterization of mice with complete SEC23A deficiency and the genetic interactions of *Sec23a* null allele with the previously reported *Sec23b* null allele^14^. SEC23A-deficient animals die during mid-embryogenesis with defects in neural tube closure and extraembryonic membrane formation. Secretion defects of multiple types of collagen are observed in SEC23A-deficient embryos, but not in SEC23B-deficient embryos.

## Materials and Methods

### Generation of SEC23A deficient mice

An ES cell line (RRE297) was obtained from BayGenomics, which contained a gene-trap insertion in intron 2 of the *Sec23a* gene. Chimeric mice were generated at the University of Michigan Transgenic Core facility as previously described^19^. Germ-line transmission was achieved by mating the male chimeric mice with C57BL/6J female mice. All mice had free access to food and water and were kept in cages in a 12-h light/dark cycle. All animal experimental protocols were approved by the Institutional Animal Care and Use Committees at the University of Michigan and the Cleveland Clinic, and carried out in accordance with the approved guidelines.

### Genotyping

Genotyping was carried out by a three-primer PCR assay of genomic DNA prepared from either tail clippings of pups or embryonic tissues. A 5′ primer (Sec23a-F2) upstream of the insertion site was combined with two 3′ primers, located downstream of the insertion site in intron 2 (Sec23a-R2) and at the 5′ end of the pGT2lxf targeting vector sequence (V20), to generate different-sized PCR products from the WT (442 bp) and the targeted allele (338 bp). Primer sequences are shown in Table S1.

### Analysis of embryos

Intercrosses of heterozygote mice were performed using mice that were backcrossed with C57BL/6J mice for 10 or more generations. The day on which the vaginal plug was detected was considered to be day 0.5 post-coitum (designated E0.5). Pregnant females were sacrificed at different days post-coitum and the embryos removed for analysis. Images were captured using a Leica camera coupled to a dissecting microscope.

### Cell culture

Mouse embryonic fibroblasts (MEFs) were prepared from E11.5 embryos as previously described^19^. MEFs were maintained in DMEM supplemented with 10% fetal bovine serum (FBS), 100 units ml^–1^ penicillin and 100 units ml^–1^ streptomycin at 37 °C in 5% CO_2_. The micromass culture protocol was modified from previous publication^20^. Briefly, mesenchymal cells were prepared from the limbs of E11.5 mice and digested with 0.5% trypsin and 0.5 mM EDTA. A total of 2 × 10^5^ cells ml^–1^ were plated and maintained in DMEM plus Ham’s F-12 nutrient mixture supplemented with ascorbic acid (100 μg ml^–1^) and β-glycerophosphate (5 mM).

### Antibodies

The primary antibodies against different collagen types used in this study were: rabbit polyclonal anti-collagen I (MD Bioproducts, catalog numbe 203002), rabbit polyclonal anti-collagen II (Acris Antibodies, catalog number R1036), rabbit polyclonal anti-collagen III (Abcam, catalog number AB7778) and rabbit polyclonal anti-collagen IV (Millipore; catalog number AB756P). According to manufacturers, all antibodies against a specific collagen type have minimal crossreactivity with other collagen types. Other antibodies used include: mouse monoclonal anti-KDEL (MBL; 1:100), mouse monoclonal anti-PDI (Assay Designs; 1:200), rabbit polyclonal anti-Ki67 (Abcam; 1:500), rabbit polyclonal anti-fibronectin (Abcam; 1:100), mouse monoclonal anti-eIF2α and rabbit polyclonal anti-p-eIF2α (Cell Signaling; 1:1000), mouse monoclonal anti-GRP78 (MBL; 1:1000), mouse monoclonal anti-GAPDH (Sigma; 1:1000) and β-actin (Sigma; 1:10000). Specific SEC23A and SEC23B polyclonal antibodies were described previously^14^.

### Histological and immunofluorescence analyses

Tissues were fixed in 10% neutral buffered formalin solution (Fisher Scientific), embedded in paraffin, and cut into 5-μm–thick sections before hematoxylin and eosin (H&E) staining. β-galactosidase staining of whole mount embryos and tissue frozen sections was as described previously^19^. Images were visualized and captured with an Axioplan2 imaging (Zeiss) microscope.

For immunofluorescence analysis, tissues were fixed in 4% paraformaldehyde, washed and incubated in 30% sucrose, before cryo-embedding. Sagittal, transverse and coronal 5-μm-thick sections or fixed MEFs were blocked in 5% BSA, permeabilized in 0.3% Triton X-100, and then incubated overnight with the primary antibodies. Fluorescent secondary antibodies conjugated with Alexa 488 or Alexa 594 (Invitrogen) was used for signal detection. Cellular nuclei were counterstained with 4,6-diamidino-2-phenylindole (Dapi). Sections were then examined under an inverted fluorescence microscope (Leica). In cell death experiments, apoptotic cells in embryonic frozen sections were detected by the TUNEL (TdT mediated dUTP nick end labeling) assay using a fluorescein-based detection kit (*in situ* death detection kit, Roche) according to manufacturer’s instructions. For Ki67 staining, quantification of the fluorescent Ki-67 signal was done using the NIH ImageJ software, by first converting images to 8-bit, then subtracting background and adjusting threshold range to 17–255 21. Signal intensities were enumerated from similar-size fields (6 fields for each sample) of different images for comparisons.

### Electron microscopy

Small pieces of skin on midbrain and yolk sac were fixed in 2.5% glutaraldehyde and 4% formaldehyde for 24 h, followed by post fixation in 1% osmium tetroxide for 1 h. After *en bloc* staining and dehydration in a graded ethanol series, samples embedded in eponate 12 medium (tell Pella Inc). Ultrathin sections (85nm) were doubly stained with 2% uranyl acetate and 1% lead citrate, and then observed using a PhilpsCM12 transmission electron microscope at an accelerating voltage of 60 kV.

### Alcian blue staining

For cartilage staining, E11.5 embryos were fixed in Bouin’s solution. After washing in 70% ethanol, the embryos were equilibrated in 5% acetic acid (2 × 1 hour). The embryos were stained in 0.05% Alcian blue in 5% acetic acid for 2 h, followed by two washes in 5% acetic acid (1 h each), and then cleared in methanol for 2 h before stored in 1:2 mixture of benzyl alcohol and benzyl benzoate. For micromass cultures, cells were fixed in cold methanol, and washed with HCl (0.1 N; pH 1.0). After staining in 1% Alcian blue, cartilage nodules were counted under a dissecting microscope.

### RNA preparation and real-time reverse transcriptase (RT)-PCR

Total RNA was extracted using the Trizol reagent (Invitrogen) and the RNeasy Micro Kit (Qiagen). RNA quantity and purity were determined by a Nanodrop spectrophotometer. The total RNA (1 μg) from each sample was reverse transcribed into cDNA using the iScript cDNA Synthesis Kit (Biorad), according to the manufacturer’s instructions. SYBR green based quantitative PCR reactions were performed in a Bio-Rad CFX96 Real Time PCR Detection System. Reaction specificity was determined by product melting curves. Relative gene expression was calculated by the 2^−ΔCt^ method using *Gapdh* or *Atcb* as reference genes.

### Statistical Analysis

Student’s t-test was used to assess the significance of differences between two groups of data (p < 0.05 is deemed significant). The CHITEST was used to evaluate the significance of difference between the expected and observed genotype distributions.

## Results

### Embryonic lethality of SEC23A-deficient mice

**Figure 1:**
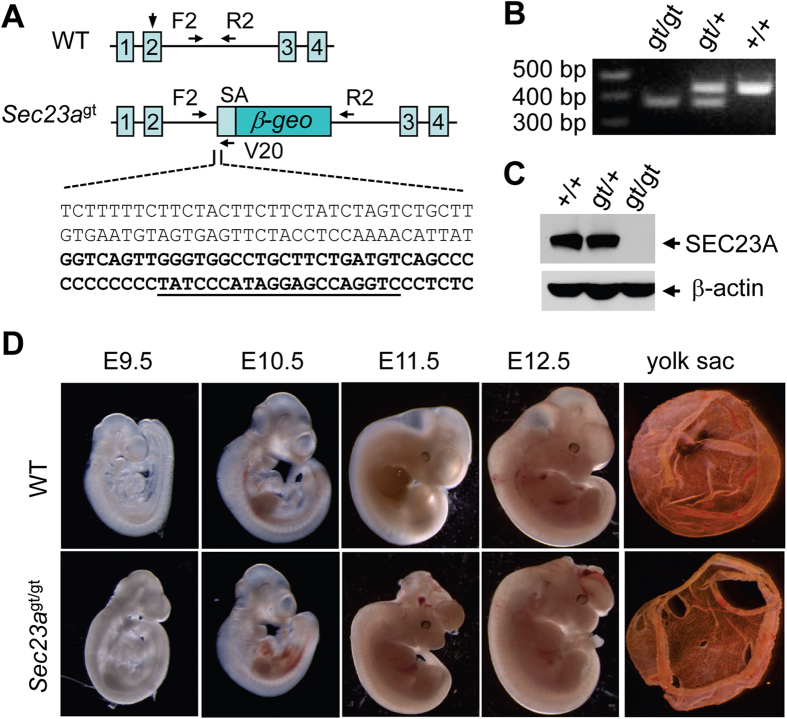
Characterization of mouse embryos with the *Sec23a* genetrap allele. (**A**) Schematic diagram of the *Sec23a* genetrap allele. Arrow head indicates the location of the initiation codon. Arrows represent locations of genotyping primers. The DNA sequence represents gene trap insertion junction in intron 2. The 5′ sequence of the *En2* intron of the genetrap cassette is shown in bold type. Primer sequence of V20 is underlined. SA, splice acceptor cassette. (**B**) Three-primer PCR genotyping results. (**C**) Western blot analysis of lysates of WT, *Sec23a*^gt/+^ and *Sec23a*^gt/gt^ cells. (**D**) Morphology of WT and *Sec23a*^gt/gt^ embryos at different time points during development and yolk sacs of E11.5 embryos. SEC23A null embryos appear normal at E9.5 and became slightly smaller in size at E10.5. At E11.5, nurotube opening starts to appear in the head of null embryos. The majority of null embryos die by E12.5 and all of them developed neural tube opening.

PCR analysis of the ES cell clone RRE229 identified the gene-trap insertion site in intron 2 of *Sec23a* (Fig. 1A). The resulting fusion transcript of the first two exons of *Sec23a* (encoding the first 73 amino acids) with the *β-Geo* cassette produces a chimeric protein missing over 90% of SEC23A, which is expected to be nonfunctional. We developed a three-primer genotyping protocol to distinguish the WT and the gene-trap alleles in a single PCR reaction (Fig. 1B). Success of the genetrap strategy relies on efficient fusion of the *β-Geo* cassette with the preceding exon of the target gene. To quantitate the residual normally spliced transcript in *Sec23a*^gt/gt^ cells, we isolated RNA from WT and *Sec23a*^gt/gt^ embryonic fibroblasts and performed real-time RT-PCR using primers spanning the exon 2–3 junction of *Sec23a* (RT2-S, located in exon 2, and RT-2AS, located in exon 4; Supplement file 1). Results show that the amount of normally spliced *Sec23a* mRNA is less than 8,000 fold lower in *Sec23a*^gt/gt^ cells than in the WT cells (data not shown). Consistently, Western blot analysis using an antibody recognizing an internal epitope near the C-terminus^14^ detected no SEC23A protein in *Sec23a*^gt/gt^ cell lysates (Fig. 1C). Intercrosses of heterozygous mice produced no homozygous gene-trap mice at the time of genotyping (~14 days after birth). Further observations demonstrated that the majority of *Sec23a*^gt/gt^ embryos died between E11.5 and E12.5 (Table 1). The *Sec23a*^gt/gt^ embryos appeared morphologically normal at E9.5-E10.5. Approximately half of live *Sec23a*^gt/gt^ embryos at E11.5 were found to have a neural tube opening at midbrain, with an exencephaly phenotype (Fig. 1D). By E12.5, all surviving (with heart beat) and dead *Sec23a*^gt/gt^ embryos had neural tube openings.

### Characterization of the neural tube phenotype of *Sec23a* ^gt/gt^ embryos

**Figure 2:**
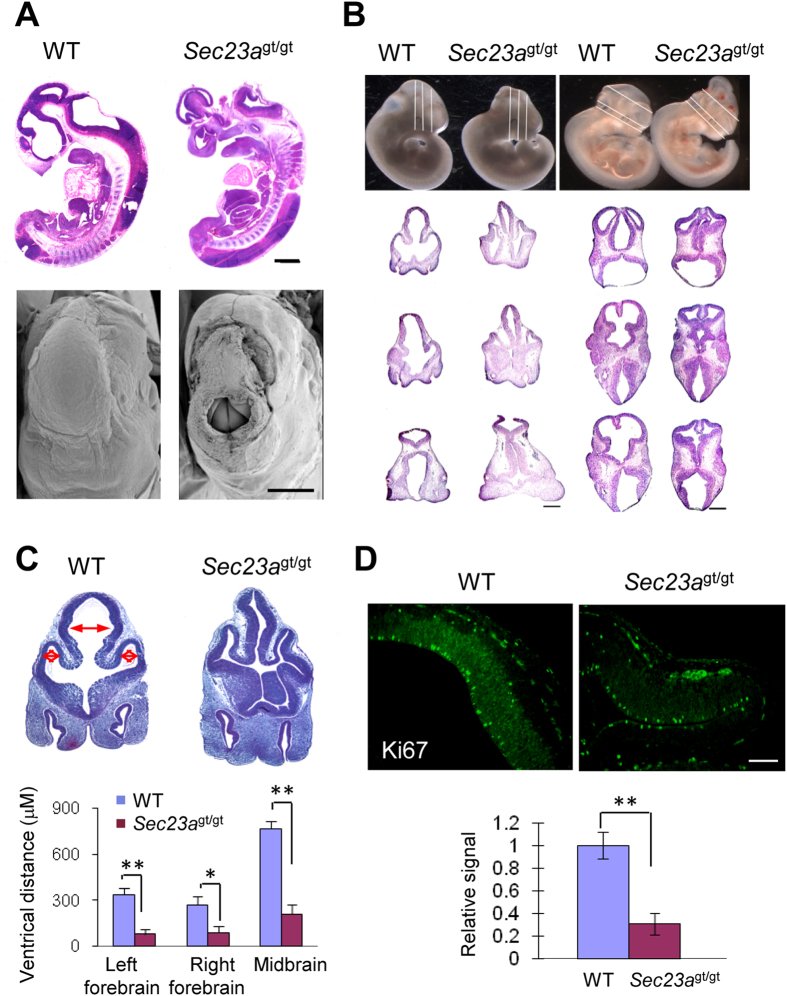
Characterization of the neural tube opening phenotype. (**A**) H&E staining of the sagittal sections and scanning electron microscopic images of E11.5 embryos showed neural tube openings at midbrain in *Sec23a*^*gt/gt*^ embryos. Bars, 500 μm. (**B**) Collapse of telencephalic vesicles and compressed brains of *Sec23a*^*gt/gt*^ versus WT embryos at E11.5. H&E-stained coronal and transverse head sections of WT and *Sec23a*^*gt/gt*^ embryos are shown. Lines on the top panel illustrate the plains of sectioning. (**C**) Quantification of the widest left to right distance of forebrains and midbrain ventricles in coronal section (μM), as depicted by red double arrows in E11.5 WT and *Sec23a*^*gt/gt*^ embryos. (**D**) Immunofluorescence staining of Ki67 in WT and *Sec23a*^*gt/gt*^ embryos on transverse sections of E11.5 cephalic neural folds in prospective forebrain and midbrain region. Quantitative analysis of Ki67 immunofluorescence staining is shown in the bar graph. Values represent means ± SD of at least three different experiments from three mice per group. **p < 0.001. Scale Bar, 200 μm.

H&E stained sections and scanning electron microscopy images of whole embryos revealed that neural tube openings in *Sec23a*^gt/gt^ embryos reached the ventricles of the midbrain (Fig. 2A). Hemorrhage was also noticeable on the head near the neural tube opening (Figs 1D and 2B). No other gross abnormalities were noted in serial sections of whole embryos. Serial coronal and transverse sections of the head revealed severe brain compression and the collapse of ventricles in *Sec23a*^gt/gt^ embryos (Fig. 2B). By measuring the widest distance of the brain ventricles in E11.5 embryos, we found that both midbrain and forebrain ventricles of *Sec23a*^gt/gt^ embryos were smaller than WT controls (Fig. 2C). We further performed a series of experiments to examine the state of cell proliferation and programmed cell death in the WT and *Sec23a*^gt/gt^ embryos. TUNEL staining demonstrated that there was no significant difference in the number of positive cells in the neural epithelium of WT and *Sec23a*^gt/gt^ embryos (data not shown). Staining with the proliferation marker Ki67 detected much weaker signals in neural epithelium of *Sec23a*^gt/gt^ embryos compared to WT embryos (Fig. 2D), suggesting attenuated cell proliferation in brains of *Sec23a*^gt/gt^ embryos with exencephaly.

### Impaired collagen I and III secretion in *Sec23a* ^*gt/gt*^ embryos

**Figure 3:**
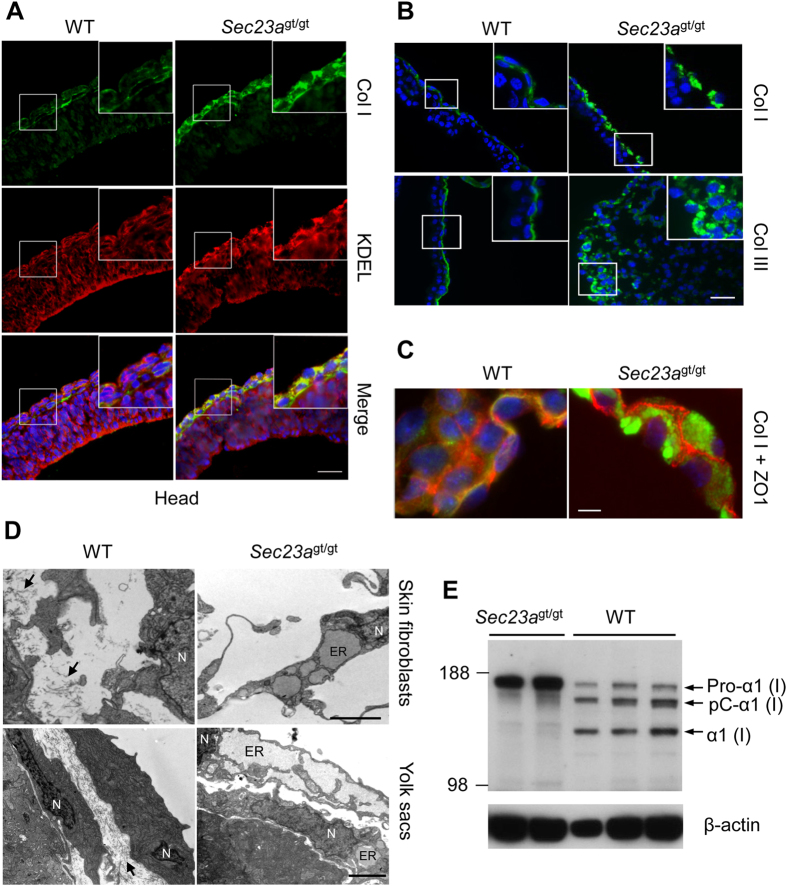
Collagen secretion defect in fibroblasts of head and extracellular membranes. (**A**) Immunofluorescence analysis of Col I in the head skin of E11.5 embryos. Col I (green) was co-stained with KDEL (red), an ER marker. Nuclei were visualized by DAPI staining (blue). Contrary to the extracellular staining pattern in the WT embryos, Col I primarily co-localizes with KDEL in *Sec23a*^*gt/gt*^ embryos. Scale bar, 100 μm. (**B**) Immunofluorescence staining of Col I in yolk sac of E11.5 embryos. Scale bar, 50 μm. (**C**) Col I (green) was co-stained with ZO1 (red), tight junction protein. Scale bar, 10 μm. (**D**) Electron microscopy images of fibroblasts in the head skin and yolk sacs of E11.5 embryos. Arrows point to collagen fibers. N, nucleus. Scale bars, 2 μm. (**E**) Collagen processing defects in *Sec23a*^*gt/gt*^ yolk sac. Equal amounts of yolk sac extracts were immunoblotted for Col I. The three forms of the α(I) chain of Col I recognized by the antibody are indicated as follows: Pro-α1(I), unprocessed form with both N- and C-propeptides; pC, Col I with the N-propeptide cleaved; and α1(I), fully processed α(I) band^63^.

Previous work on human skin fibroblasts with CLSD mutations suggests collagen secretion defects^8^,^10^. Type I collagen (Col I) is abundantly expressed in connective tissues, such as skin, organ sheath, and blood vessels^22^,^23^. Type III collagen (Col III) is a main component of reticular fibers and commonly found alongside Col I. We carried out immunohistochemical staining on the sagittal sections of embryos. In WT embryos, Col I staining was mainly observed in the extracellular space of the fibroblasts of the primitive skin that covers the embryonic head. In contrast, in *Sec23a*^gt/gt^ embryo, Col I staining occurred in the intracellular space (Fig. 3A). Double staining with an anti-KDEL antibody, which marks the ER, showed that the intracellular Col I is localized to the ER (Fig. 3A). Similar results were obtained with Col III staining (Fig. 3B, Figure S1). These results suggest Col I and Col III secretion defects in skin fibroblasts in *Sec23a*^gt/gt^ embryos.

We also noted that, upon dissection, a majority of yolk sacs of *Sec23a*^gt/gt^ embryos are broken, while most WT yolk sacs remain intact (Fig. 1D). Moreover, many *Sec23a*^gt/gt^ embryonic amnions at E11.5 did not completely encase the embryo. Both yolk sac and amnion consist of an outer endoderm layer and an inner mesoderm layer. Collagens are major components of the ECM in the mesoderm layer^24^. To test whether collagen secretion defects occur in these extraembryonic membranes, we performed immunofluorescence staining on frozen sections of E11.5 yolk sac with Col I and Col III antibodies. The results showed that both Col I and Col III accumulate intracellularly in mesothelial cells of *Sec23a*^gt/gt^ embryos, while they mainly reside in the extracellular space in WT embryos (Fig. 3B). Intracellullar accumulation of Col I was apparent in double staining with anti-Col I and anti-ZO1 (a tight junction protein) antibodies (Fig. 3C). Intracellular accumulation of Col I and Col III was also observed in multiple other tissues, including amnion, liver, heart, blood vessel walls, and mesenchyme in *Sec23a*^gt/gt^ embryos (Figure S1).

The ultrastructures of collagen-secreting cells in E11.5 embryos were further examined by transmission EM. Fibroblasts in the outer layer of WT embryonic head and the mesoderm layer of the yolk sac contain the normal cisternae structure of the rough ER, and collagen fibers can be detected in extracellular spaces (Fig. 3D, arrows). In contrast, in *Sec23a*^gt/gt^ embryonic head and yolk sac, collagen-producing cells contain grossly distended ER and no collagen fibers were detected in extracellular spaces (Fig. 3D). We further analyzed protein extracts from WT and *Sec23a*^gt/gt^ yolk sacs for Col I by immunoblotting (Fig. 3E). In WT tissues, both pro-collagen, which represents the intracellular protein, and two processed forms, which represent secreted proteins, were detected. The majority of Col I exists as processed forms. However, in *Sec23a*^gt/gt^ tissues, pro-collagen was the predominant form, with little processed forms of Col I detected. Similar results were obtained in amnion (Figure S2). The secretion defects of Col I and Col III are associated with lower mRNA levels of *Col1a1* and *Col3a1* in *Sec23a*^gt/gt^ yolk sac and amnion, suggesting a negative feedback regulation of collagen genes (Figure S3). Taken together, these results indicate that Col I and Col III secretion defects occur in multiple tissues of *Sec23a*^gt/gt^ embryos.

### Impaired secretion of other collagen types in *Sec23a* ^gt/gt^ embryos

**Figure 4:**
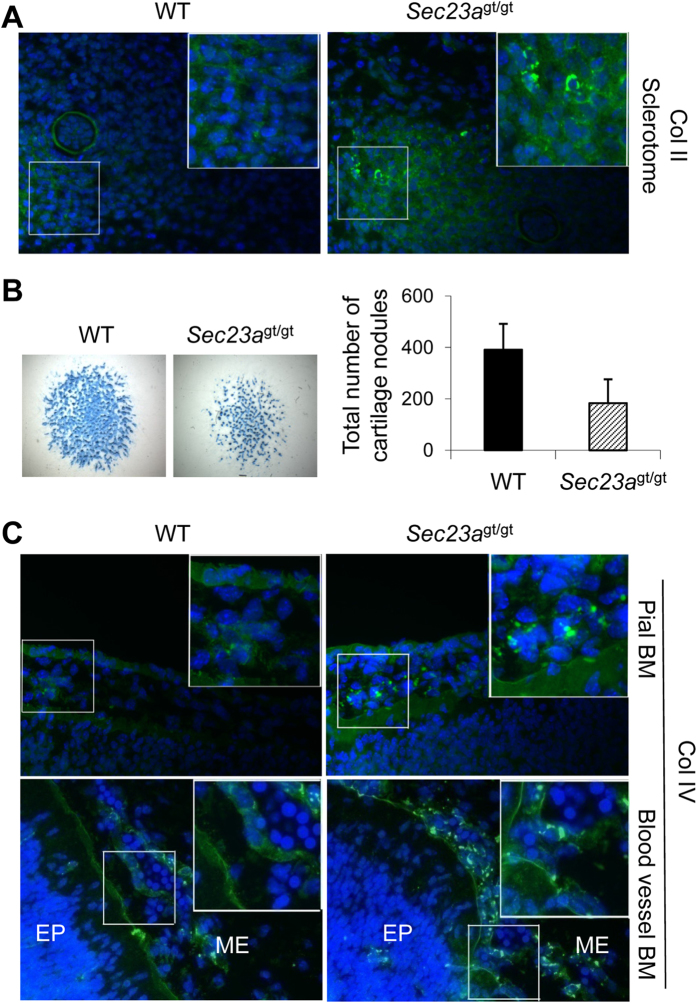
Col II and Col IV secretion defects in *Sec23a*^*gt/gt*^ embryos. (**A**) Immunofluorescence staining of Col II (green) on transverse section of E11.5 WT and *Sec23a*^*gt/gt*^ embryos. Nuclei were visualized by DAPI staining (blue). Col II accumulated in the procartilaginous cells in sclerotome of *Sec23a*^*gt/gt*^ embryos. Scale bar, 100 μm. (**B**) Col II secretion defect of *Sec23a*^*gt/gt*^ embryos. Mesenchymal cells were maintained as micromass cultures for 7 days. Cells were stained with Alcian blue for cartilage matrix proteins. *Sec23a*^*gt/gt*^ cells showed significantly lower ECM protein secretion than WT cells. Scale bar, 1 mm. ECM proteins were quantified by optical absorbance. The number of Alcian blue-stained nodules of day 7 micromass cultures was counted manually. Data are mean ± SD, *n *= 5 (*P < 0.05, **P < 0.01 Student’s *t*-test). (**C**) Immunofluorescence staining indicates that Col IV accumulation occurs in collagen-producing cells in pial basement membranes and blood vessel basement membranes. ME, mesenchyme; EP, epithelium. Scale bar, 50 μm.

Type II collagen (Col II) is the major protein component of cartilages. Mouse embryos at E11.5 are just beginning to form cartilages. To determine if SEC23A deficiency interferes with mouse chondrogenesis, we performed Alcian blue staining of E11.5 WT and *Sec23a*^gt/gt^ embryos with no neural tube phenotype and observed no obvious morphologic differences in nascent embryonic cartilages between the two groups (Figure S4). To determine whether WT and *Sec23a*^gt/gt^ embryos have Col II secretion differences, we conducted immunofluorescence analysis for Col II in sclerotome areas^25^ of E11.5 embryos. As shown in Fig. 4A, instead of extracellular staining in WT embryos, Col II accumulates in the procartilaginous cells in *Sec23a*^gt/gt^ embryos. We further investigated the effects of SEC23A deficiency on cartilage formation using micromass culture of mesenchymal cells prepared from E11.5 embryos. The total cartilage nodule numbers are significantly reduced in micromass cultures from *Sec23a*^gt/gt^ cells compared to WT cells (Fig. 4B). These results suggest that further perturbation of cartilage formation would have been seen if *Sec23a*^gt/gt^ embryos had survived beyond E12.5.

We also tested the secretion of the type IV collagen (Col IV), which is a network-forming collagen and the main component of basement membranes (BMs). BMs separate epithelial (or endothelial) cells from mesenchymal tissues and maintain the function and morphology of epithelial cells^26^. Similar to other collagen types tested, immunofluorescence staining showed that Col IV accumulated within cells in the pial BM in the brain and in the blood vessel BM in *Sec23a*^gt/gt^ embryos, in contrast to the extracellular staining of Col IV in WT embryos (Fig. 4C). Taken together, our results suggest broad secretion defects of multiple collagen species in different tissues.

### Increased apoptosis in extraembryonic membranes of *Sec23a* ^gt/gt^ embryos

**Figure 5:**
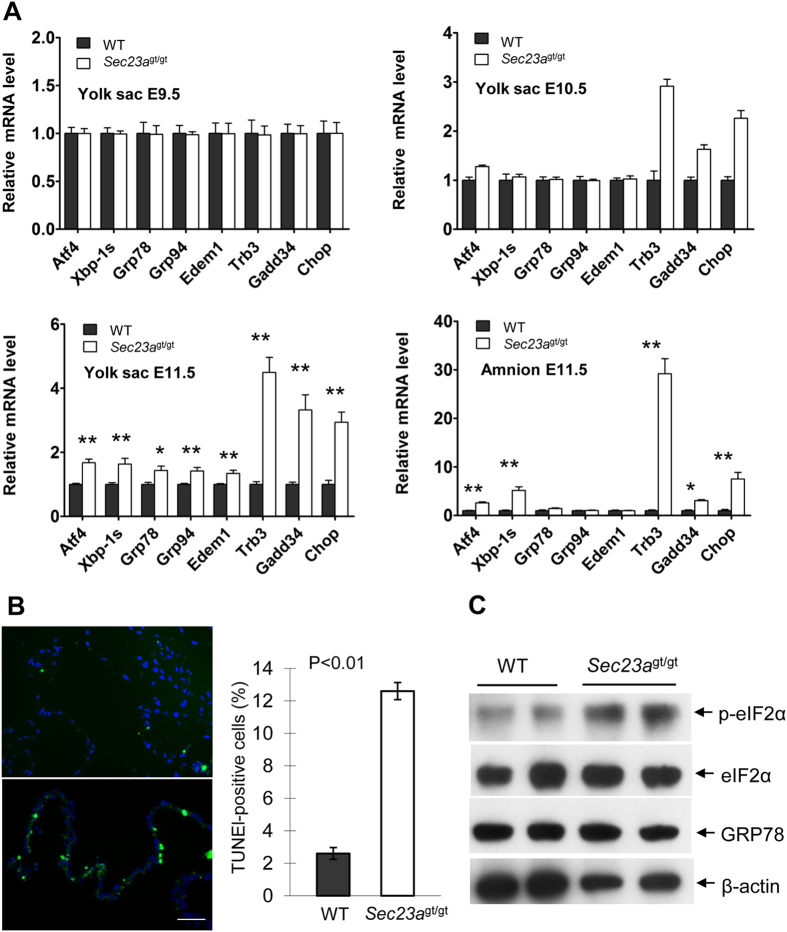
Accumulation of collagens in the ER leads to activation of the pro-apoptotic branch of UPR pathway. (**A**) Real-time RT-PCR quantification of select UPR genes in E9.5–E11.5 yolk sac and E11.5 amnion. Expression of the GAPDH gene was used as a reference. Data are mean ± SD. Asterisks indicate statistically significant difference between WT and *Sec23a*^*gt/gt*^ samples (*P < 0.05, **P < 0.01). (**B**) TUNEL staining (green) was performed for E11.5 amnion. Nuclei were visualized by DAPI staining (blue). TUNEL-positive cells were quantified as percentages of total cell numbers. (**C**) Activation of the PERK pathway in *Sec23a*^*gt/gt*^ yolk sacs. Equal amounts of extracts from E11.5 yolk sac were immunoblotted for eIF2α, the phospohorylated eIF2α (p-eIF2α), GRP78 and β-actin.

To determine whether specific unfolded protein response (UPR) pathways^27^,^28^ are activated in these cells, we measured expression levels of representative UPR genes in WT and *Sec23a*^gt/gt^ yolk sacs and amnion at different time points (E9.5, E10.5, E11.5) during embryogenesis by RT-PCR (Fig. 5A). We observed progressively more pronounced increases in expression of certain UPR genes in *Sec23a*^gt/gt^ yolk sacs compared to WT yolk sacs. In particular, expression of genes that are associated with ER stress-induced apoptosis was increasingly elevated in *Sec23a*^gt/gt^ yolk sacs from E9.5 to E11.5. Similarly, UPR genes that are associated with apoptosis are drastically overexpressed in E11.5 *Sec23a*^gt/gt^ amnion (Fig. 5A). Apoptosis serves to remove severely damaged cells during ER stress. TUNEL staining revealed markedly increased number of apoptotic cells in *Sec23a*^gt/gt^ amnion at E11.5 (Fig. 5B). The increased *Atf4, Ddit3* (encoding CHOP) and *Trb3* (a target of CHOP/ATF4)^29^ expression suggests that the PERK pathway of the UPR^27^,^28^ was preferentially activated. In support of this hypothesis, the levels of phosphorylated eukaryotic translational initiation factor 2α (p-eIF2α), which is associated with translational repression in the UPR^30^,^31^, was up-regulated in *Sec23a*^gt/gt^ yolk sacs (Fig. 5C). These results demonstrate that collagen accumulation in the ER of SEC23A deficient extraembryonic membranes induces ER stress and apoptosis mainly by activating the PERK pathway of the UPR.

### SEC23B deficiency does not lead to collagen secretion defects

**Figure 6:**
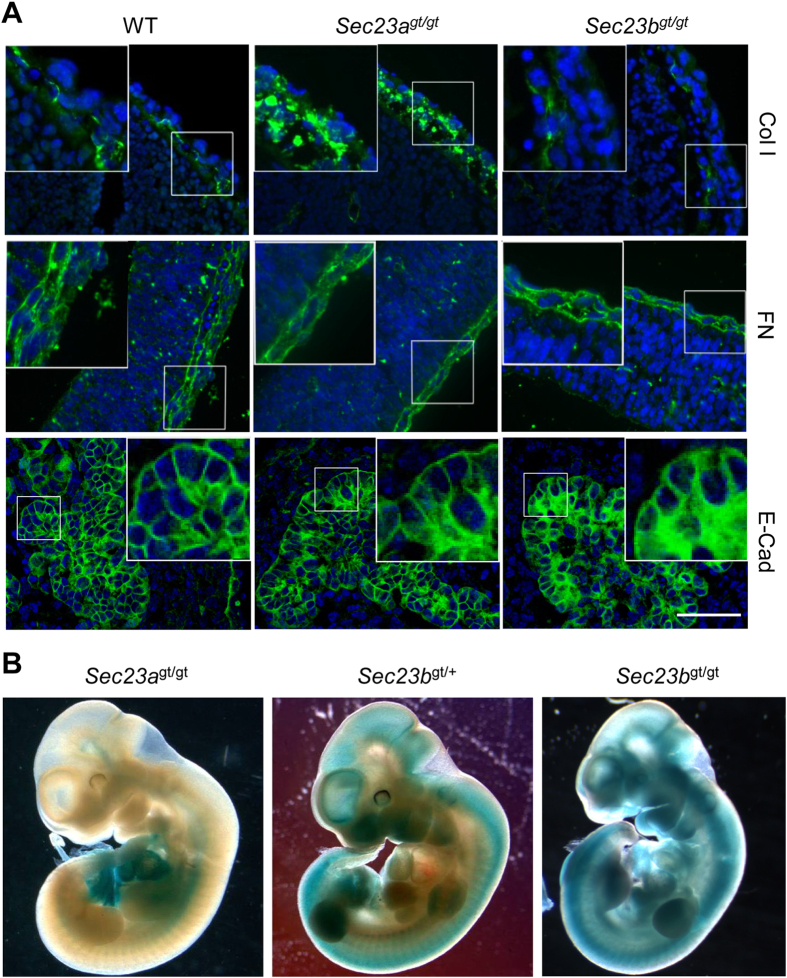
Collagen secretion is mediated by SEC23A. (**A**) Immunofluorescence staining of Col I and fibronectin (FN) in head sections and E-cadherin in pancreas sections of WT, *Sec23a*^*gt/gt*^ and *Sec23b*^*gt/gt*^ embryos at E11.5. Scale bar, 100 μm. (**B**) Whole-mount X-gal staining of *Sec23a*^gt/gt^, *Sec23a*^gt/+^ and *Sec23b*^gt/gt^ embryos at E10.5.

We further explored whether SEC23B also plays a similar role in collagen secretion. Immunofluorescence staining showed that, in contrast to the intracellular staining in *Sec23a*^gt/gt^ embryos (Fig. 6A), Col I was primarily observed in the extracellular space of the *Sec23b*^gt/gt^ embryonic connective tissues, which is indistinguishable from the staining pattern of WT tissues (Fig. 6A). Other collagen types also showed similar staining patterns between WT and *Sec23b*^gt/gt^ embryos (data not shown), suggesting that collagen secretion is not significantly affected by the absence of SEC23B. Staining pattern of fibronectin, another major extracellular matrix protein involved in fibrillar network formation^32^, is not altered in either *Sec23a*^gt/gt^ or *Sec23b*^gt/gt^ embryos (Fig. 6A).

Previously we reported accumulation of zymogens in *Sec23b*^gt/gt^ pancreatic acinar cells. We performed immunofluorescence staining of additional proteins expressed in exocrine pancreas at E11.5, a stage before zymogens begin to be expressed in these cells. We identified altered staining pattern of E-cadherin (CDH1), which is localized to the periphery of cells in WT pancreas, but shifts to the cytoplasm of *Sec23b*^gt/gt^ acinar cells (Fig. 6A). The change in CDH1 staining pattern in *Sec23b*^gt/gt^ pancreas is more evident later in development. At E15.5, CDH1 localization is distinct from the interior staining of carboxylpeptidase A (CPA) in WT acinar cells (Figure S5). In *Sec23b*^gt/gt^ pancreas, CDH1 largely co-localizes with CPA, suggesting that it remains inside the ER. β-catenin, which binds to CDH1, also undergoes similar shifts in staining pattern in *Sec23b*^gt/gt^ pancreas (Figure S5). Mislocalization of CDH1 and β-catenin is also observed in salivary glands of *Sec23b*^gt/gt^ embryos (Figure S5). Therefore, distinct groups of cargo proteins are affected by SEC23A and SEC23B deficiencies.

### Compensatory increase of one paralog of SEC23 in the absence of the second paralog

We have previously shown the relative expression levels of SEC23A and SEC23B vary in different tissues^14^. The severe phenotype of *Sec23a*^gt/gt^ embryos could be attributed to the insufficient availability of SEC23B to provide normal SEC23 function in collagen secreting cells of *Sec23a*^gt/gt^ embryos. With both gene-trap alleles of *Sec23a* and *Sec23b*, we can directly visualize the relative expression levels of these two genes in developing embryos with X-gal staining. Whole-mount X-gal staining of E10.5 embryos of *Sec23b*^gt/+^ and *Sec23a*^gt/gt^ revealed a largely complementary expression patterns of these two paralogs (Fig. 6B).

**Figure 7:**
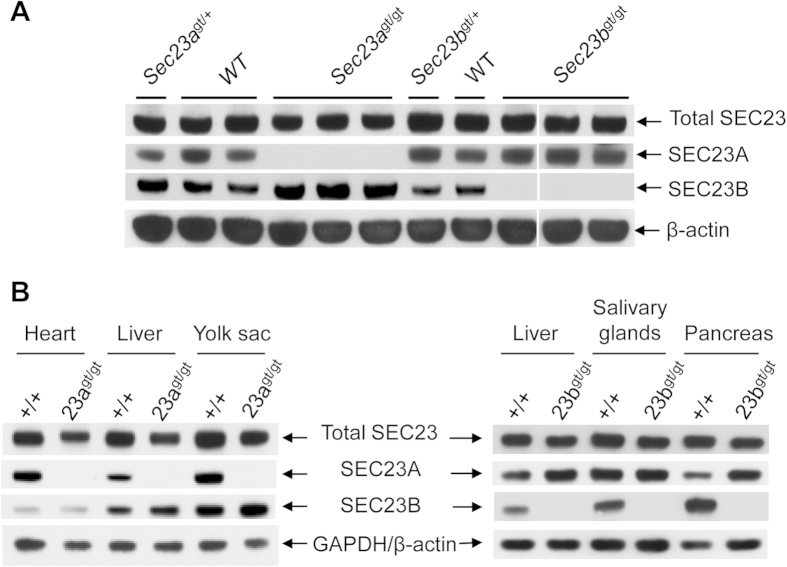
Comparison of SEC23A, SEC23B and total SEC23 in WT, *Sec23a*^*gt/gt*^ and *Sec23b*^*gt/gt*^ cells and tissues. (**A**) Comparison of SEC23A, SEC23B and total SEC23 in MEFs. Equal amounts of cell lysates from MEFs of different genotypes were immunoblotted for total SEC23, SEC23A, SEC23B and β-actin. (**B**) Comparison of SEC23A, SEC23B and total SEC23 in MEFs. Equal amounts of extracts from different E11.5 embryonic tissues of different genotypes were immunoblotted for total SEC23, SEC23A, SEC23B and β-actin/GAPDH.

Does deficiency of one SEC23 paralog affects the level of the remaining paralog? We compared SEC23A, SEC23B and total SEC23 levels in MEFs of WT, *Sec23a*^gt/+^, *Sec23a*^gt/gt^, *Sec23b*^gt/+^ and *Sec23b*^gt/gt^ genotypes, by Western blot analysis using paralog-specific antibodies and an antibody recognizing a peptide sequence common to both SEC23A and SEC23B. Results show elevated SEC23B level in *Sec23a*^gt/gt^ cells (and to a lesser extent in *Sec23a*^gt/+^ cells) and elevated SEC23A level in *Sec23b*^gt/gt^ cells (and to a lesser extent in *Sec23a*^gt/+^ cells), compared to WT cells (Fig. 7A). As a result, the overall amounts of SEC23 proteins are only moderately decreased in *Sec23a*^gt/gt^ and *Sec23b*^gt/gt^ cells. Compensatory increases in SEC23B and SEC23A levels are also seen in *Sec23a*^gt/gt^ and *Sec23b*^gt/gt^ tissues respectively (Fig. 7B). The *Sec23a* and *Sec23b* mRNA levels are not significantly increased in *Sec23b*^gt/gt^ and *Sec23a*^gt/gt^ MEFs respectively (data not shown), suggesting that posttranslational regulation is the main mechanism of increases in SEC23A or SEC23B protein levels in these cells. However, the elevated level of the remaining paralog is apparently not sufficient to functionally compensate for the loss of the first paralog.

### SEC23 paralogs partially overlap in function *in vivo*

To test the genetic interactions between *Sec23a* and *Sec23b*, we crossed *Sec23a*^gt/+^ mice and *Sec23b*^gt/+^ mice. The expected number of double heterozygous mice was produced from the intercross, and these mice are viable and fertile, with no discernible phenotype (data not shown). Next we performed intercrosses of the double heterozygous mice with *Sec23a*^gt/+^ or *Sec23b*^gt/+^ mice (Table 2). Mating of *Sec23a*^gt/+^ with *Sec23a*^gt/+^ *Sec23b*^gt/+^ mice revealed a severe loss of *Sec23a*^gt/gt^ *Sec23b*^gt/+^ embryos at E11.5 and a partial loss at E9.5, compared to the *Sec23a*^gt/gt^ embryos. Mating of *Sec23b*^gt/+^ with *Sec23a*^gt/+^ *Sec23b*^gt/+^ mice revealed even more severe loss of *Sec23a*^gt/+^ *Sec23b*^gt/gt^ embryos, as no embryos with this genotype were identified at E18.5 and E9.5. Therefore, a further decrease in the expression of the remaining *Sec23* paralog on top of homozygous inactivation of the first paralog leads to earlier lethality of embryos. These results indicate a partial overlap of functions of SEC23A and SEC23B *in vivo*.

## Discussion

We show that complete deficiency of SEC23A results in lethality during mid-embryogenesis, indicating that SEC23A is essential for normal embryonic development. The *Sec23a*^gt/gt^ embryos develop exencephaly between E10.5 and E12.5, accompanied by compromised extraembryonic membranes. The phenotype is distinct from loss of other COPII components in mice^14^,^33^,^34^,^35^,^36^, and more severe than human CLSD, which is caused by missense mutations in SEC23A^8^,^9^, and a syndromic form of osteogenesis imperfect caused by mutations in *SEC24D*^37^. Both missense mutations of SEC23A identified in CLSD patients to date appear to result in only subtle defects in protein function. The F382L mutant is ineffective in recruiting the SEC13-SEC31 complex, while the M702V mutation activates SAR1B GTPase more than wild-type SEC23A when SEC13-SEC31 is present^10^,^11^. Our results suggest that a complete loss of SEC23A in humans likely is also lethal in the embryonic stage.

Our results show that SEC23A-mediated collagen secretion plays an important role in extraembryonic membrane formation. The extraembryonic membranes, including yolk sac and amnion, are the thin layers of tissue that surround the developing embryo. These membranes provide protection and means to transport nutrients into and wastes out of the embryo^38^. In several mouse models, the occurrence of embryonic lethality between E10.5 to E12.5 is due to defective development of the extraembryonic membranes, which become essential at this stage of gestation^39^. Col I and Col III are major components of fibrils in extraembryonic membranes and developing embryonic skin^17^,^40^. Resistance to rupture of fetal membranes is provided almost exclusively by collagens^41^. We show that both Col I and Col III accumulate in the ER of mesoderm cells and collagen fibrils in extracellular matrix are severely compromised in *Sec23a*^gt/gt^ yolk sacs.

Another prominent phenotype of *Sec23a*^gt/gt^ embryos is neural tube opening (exencephaly) observed beginning at E11.5. To our knowledge, this is the first reported example of reopening of closed neural tube during embryogenesis. Col I and Col III secretion defects in skin fibroblasts likely compromised the tensile strength of the skin tissue and result in neural tube opening at the weak point of the midbrain position in the rapidly growing embryonic head. BMs play important roles in the morphogenesis of tissues during embryonic development, separating the ectoderm and from the mesenchyme in the brain. Col IV is the main component in BMs^40^ and we have shown that it has a similar secretion deficiency as Col I and Col III. Weakening of the BM also likely contributed to the neural tube opening phenotype. Of note, collagen secretion and skull ossification defects are hallmarks of both CLSD caused by *SEC23A* mutations^8^,^9^ and osteogenesis imperfect caused by *SEC24D* mutations^37^. Interestingly, SEC24B deficiency leads to closure failure of the entire neural tube due to planar cell polarity defects resulting from defective Vangl2 trafficking^36^,^42^. The mechanism of neural tube opening in *Sec23a*^gt/gt^ embryos is likely distinct from SEC24B deficient embryos, as it is the result of reopening of a closed neural tube and the opening is limited to the midbrain region.

We also observed secretion deficiency of Col II in procartilaginous cells and in micromass cultures. This is consistent with a recent study showing that disturbed secretion of Col II was associated with decreased SEC23A expression in chondrocytes^43^. Taken together, these results indicate that SEC23A plays essential roles on the global collagen secretion and connective tissue development. Although mice deficient in several collagen types, including COL IV^44^, COL VI^45^, COL IX^46^, COL X^47^, COL XIII^48^ and COL XV^49^, are viable, defects in global collagen synthesis and secretion associated with HSP47 and TANGO1 knockout mice, as well as homozygous deletion of *Col5a1* (encoding COL V), lead to embryonic lethal phenotypes^50^,^51^,^52^. A viral insertion into the *Col1a1* gene abolished the transcription of the gene and leads to an embryonic lethal phenotype between E12 and E14^53^. For *Sec23a*^gt/gt^ embryos, global collagen secretion defects are likely the primary reason for the premature death.

Collagens accumulate in the ER and fail to undergo proper proteolytic processing in *Sec23a*^gt/gt^ embryos. The accumulation of aggregated collagen in the ER leads to selective activation of the PERK pathway of the UPR in yolk sac and amnion. This is reminiscent of the pancreas in SEC23B deficient mice, in which accumulation of zymogens in the ER lead to activation of pro-apoptotic genes in the UPR^14^. Consistent with these results, we observed increased TUNEL-positive cells in *Sec23a*^gt/gt^ amnion. Of note, *Sec23a* itself has previously been shown to be a target of an ER stress transducer, BBF2H7. Ablation of BBF2H7 leads to SEC23A deficiency and accumulation of Col II in the ER of chondrocytes^43^.

The loss of one SEC23 paralog tends to increase the amount of the remaining paralog in cells. Compensatory increases in SEC23B level result in only moderate decreases in total SEC23 levels in *Sec23a*^gt/gt^ MEFs and tissues. Apparently the increased SEC23B expression is not sufficient to compensate for the loss of SEC23A in collagen secretion. Similarly, the increase in SEC23A expression in *Sec23b*^gt/gt^ pancreas and salivary glands is not sufficient to prevent the degeneration of these organs and the mislocalization of E-cadherin. The remaining paralog plays a crucial role in the development of SEC23A null embryos to E12.5 and SEC23B null embryos to term, as more severe phenotypes were observed with haploinsufficiency of the remaining paralog (*Sec23a*^gt/gt^*/Sec23b*^gt/+^ and *Sec23a*^gt/+^*/Sec23b*^gt/gt^ embryos). A progressive loss of SEC23A was observed during erythropoiesis in humans but not in mice, potentially explaining the erythrocyte-specific phenotype of CDAII patients^54^,^55^,^56^. Craniofacial and skeletal defects were also reported in losses of activities of other COPII components in zebrafish, including *crusher* (*sec23a*)^57^, *sec23a* and *sec23b* morphant^9^,^57^, *bulldog* (*sec24d*)^58^ and *sec13* morphant^59^. However, we observed no collagen secretion defects in SEC23B null mice, likely reflecting differences in paralog usage between species.

Recent studies have also provided evidence suggesting that mouse SEC24 paralogs have developed unique functions over the course of vertebrate evolution^33^,^34^,^35^,^36^. SEC23A and SEC23B share ~85% sequence identity and thus likely overlap in functions. Differences in temporal and spatial expression patterns of SEC23A and SEC23B may explain why SEC23B cannot fully compensate for the missing SEC23A, and vice versa. Alternatively or in addition, intrinsic differences between SEC23A and SEC23B may favor the use of SEC23A for loading large and elongated cargo such as collagens into COPII vesicles. For example, SEC23A may preferentially interact with specialized adaptor proteins for collagen cargo, such as TANGO1 and cTAGE5^60^,^61^. Interestingly, the phenotype of TANGO1 knockout mice is similar to that of *Sec23*^*gt/gt*^ mice^52^, both of which have secretion defects of multiple collagen species. *Sec23a*^gt/gt^ embryos die at an earlier stage than TANGO1 knockout embryos, suggesting more severe collagen secretion defects and/or additional role of SEC23A in embryonic development. Another possibility is that SEC23A and SEC23B may have different GAP activities or affinities for other COPII components such as specific SAR1 and SEC31 paralogs, or tethering proteins such as TRAPPI^6^, which make SEC23A a more suitable molecule for the formation and maintenance of large vesicles. Finally, SEC23A and SEC23B may be differentially phosphorylated^3^, or interact with phosphorylated SEC24 and monoubiquitinated SEC31^62^ differentially, influencing COPII vesicle sizes.

## Additional Information

**How to cite this article**: Zhu, M. *et al.* Neural tube opening and abnormal extraembryonic membrane development in SEC23A deficient mice. *Sci. Rep.* **5**, 15471; doi: 10.1038/srep15471 (2015).

## Supplementary Material

Supplementary Information

## Figures and Tables

**Table 1:** Genotype distribution of embryos from intercrosses of *Sec23a*^*gt/*+^ mice.

**Expected**	**Genotype distribution**			**p value (χ**^**2**^)
	***Sec23a***^**+/+**^	***Sec23a***^***gt/*****+**^	***Sec23a***^***gt/gt***^	
	25%	50%	25%	
E9.5	24.3% (17)	52.9% (37)	22.9% (16)	>0.8
E10.5	23.6% (13)	54.5% (30)	21.8% (12)	>0.9
E11.5	25.4% (126)	53.9% (268)	20.7% (103)	<0.01
E12.5	34.6% (107)	62.5% (193)	2.9% (9)	<0.01
P14	32.1% (76)	67.9 (161)	0% (0)	<0.01

Observed numbers are listed in parentheses. Approximately 0% of *Sec23a*^*gt/gt*^ embryos at E9.5 and E10.5, 45% of *Sec23a*^*gt/gt*^ embryos at E11.5 and 100% of *Sec23a*^*gt/gt*^ embryos at E12.5 exhibit open neural tube phenotype.

**Table 2:** Genotype distribution of embryos from different intercrosses of mice with various *Sec23a* and *Sec23b* genotypes.

**Crosses**	**Genotype distribution**						**p value (χ**^**2**^)
***Sec23a***^***gt/*****+**^ **X** ***Sec23a***^***gt/*****+**^***Sec23b***^***gt/*****+**^	**+/+, +/+**	**+/+, gt/+**	**gt/+. +/+**	**gt/+, gt/+**	**gt/gt, +/+**	**gt/gt, gt/+**	
Expected	12.50%	12.50%	25%	25%	12.50%	12.50%	
E9.5	18.9% (17)	12.2% (11)	24.4% (22)	26.7% (24)	12.2% (11)	5.6% (5)	>0.1
E11.5	15.0% (9)	16.7% (10)	28.3% (17)	26.7% (16)	13.3% (8)	0% (0)	<0.01
***Sec23b***^***gt/*****+**^ **X** ***Sec23a***^***gt/*****+**^***Sec23b***^***gt/*****+**^	**+/+, +/+**	**+/+, gt/+**	**+/+. gt/gt**	**gt/+, +/+**	**gt/+, gt/+**	**gt/+, gt/gt**	
Expected	12.50%	25%	12.50%	12.50%	25%	12.50%	
E9.5	12.8% (5)	20.5% (8)	17.9% (7)	20.5% (8)	28.2% (11)	0% (0)	<0.01
E18.5	21.8% (12)	27.3% (15)	16.4% (9)	10.9% (6)	23.6% (13)	0% (0)	<0.01

Observed numbers are listed in parentheses. For genotype designation in the table, *Sec23a* genotypes are followed by *Sec23b* genotypes. The Chi test was done by comparing observed genotype distributions (homozygote of one *Sec23* ortholog vs. homozygous of one *Sec23* ortholog plus heterozygote of the second ortholog) in the shaded cells with the expected 1:1 genotype distribution.
