# Supplementary material for: Neural tube opening and abnormal extraembryonic membrane development in SEC23A deficient mice
Source: Sci Rep. 2015 Oct 23;5:15471. doi: 10.1038/srep15471 (PMC4616029; doi:10.1038/srep15471)
Supplement: Supplementary Information [file srep15471-s1.pdf]

Neurotube opening and abnormal extraembryonic membrane development in SEC23A deficient mice

Min Zhu[1,2], Jiayi Tao[2], Matthew P. Vasievich[3], Wei Wei[2], Guojing Zhu[3], Rami N. Khoriaty[3], Bin Zhang[2]¶

Table S1. Primers used in the study.

Primer Name	Sequence (5' TO 3')
Sec23a-F2	TTGGTCATAATTGAGTTGGTGTG
Sec23a-R2	CAACCACAGGAAAAGGTTGC
V20	GACCTGGCTCCTATGGGATA
Sec23a-RT2-S	GTGCAGTTTTGAATCCTTTATG
Sec23a-RT2-AS	GGGACCACGCAGAACTACAT
Col1a1-S	CCGTGCTTCTCAGAACATCA
Col1a1-AS	GAGCAGCCATCGACTAGGAC
Col3a1-S	GTCCACGAGGTGACAAAGGT
Col3a1-AS	GATGCCCCACTTGTTCCATCT

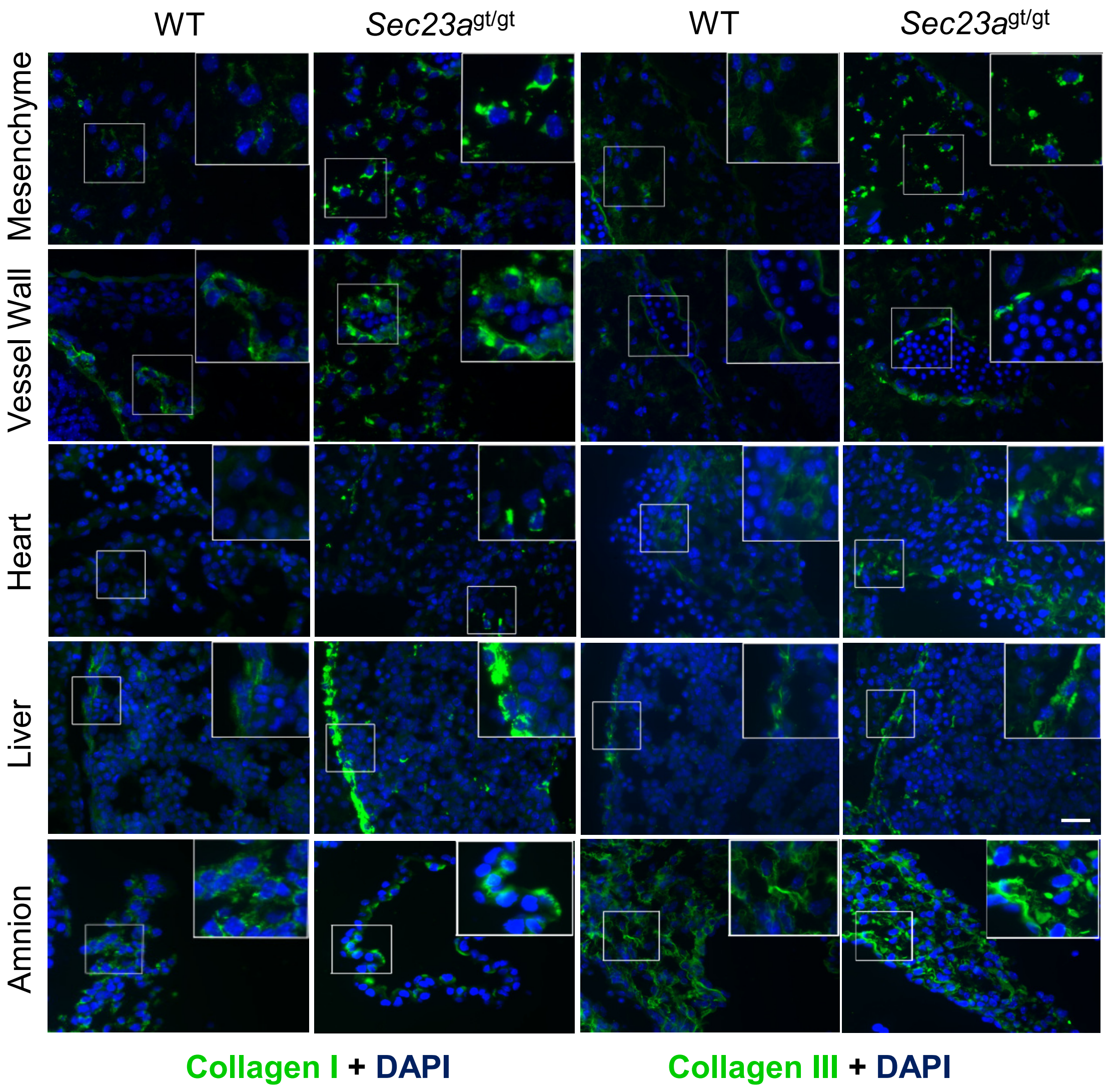

Figure S1. Col I and Col III secretion defects occur in collagen-producing cells from multiple tissues. Scale bar, 50 μm.

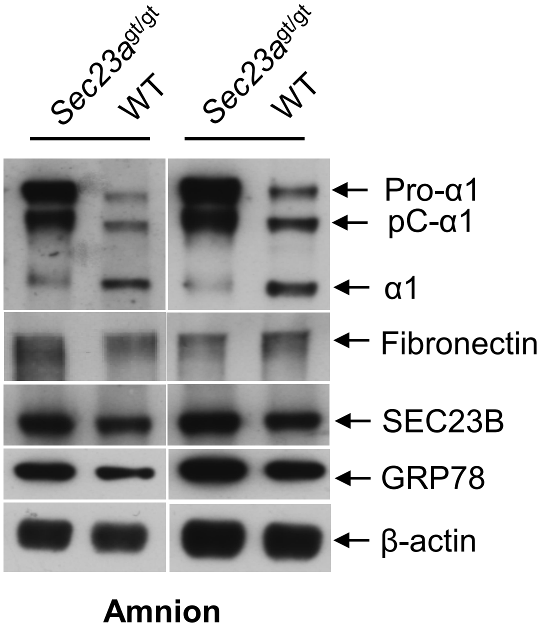

Figure S2. Col I processing is defective in *Sec23a*$^{gt/gt}$ amnion. Two pairs of WT and *Sec23a*$^{gt/gt}$ amnion were analyzed by Western blotting with the indicated antibodies.

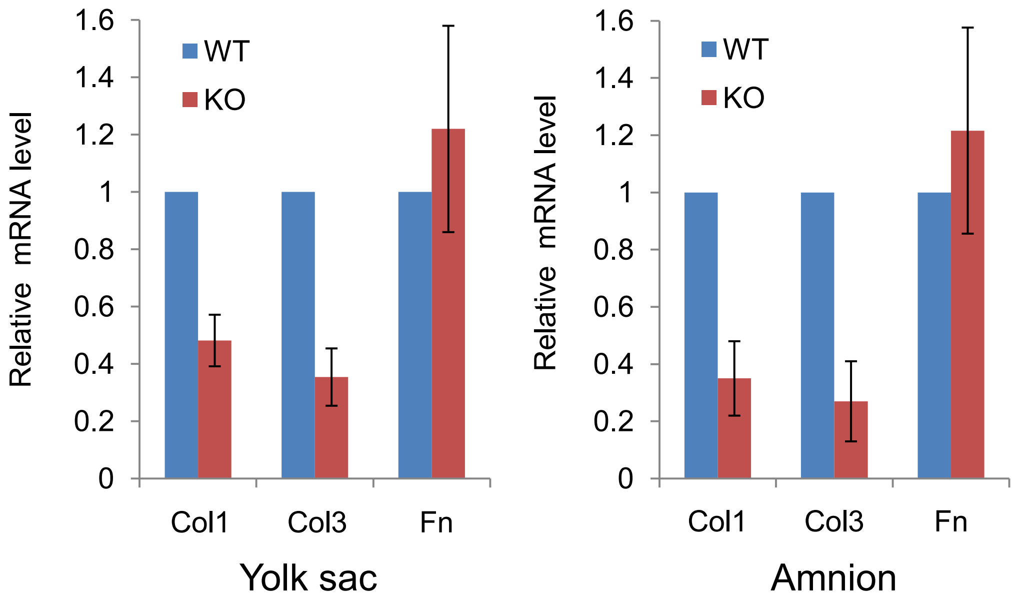

Figure S3. Col I and Col III messenger RNA levels are decreased in *Sec23a*$^{gt/gt}$ yolk sac and amnion. Semi-quantitative real-time PCR was performed in reverse transcribed RNA of WT and *Sec23a*$^{gt/gt}$ yolk sac and amnion to compare *Col1a1* and *Col3a1* and *Fn1* RNA levels.

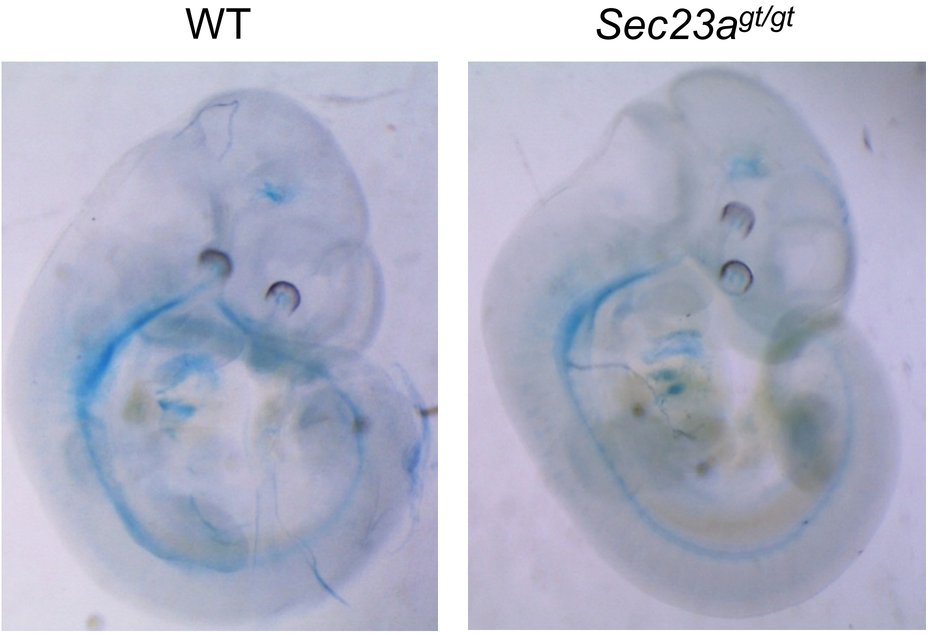

Figure S4. Whole-mount Alcian blue staining reveals no obvious differences in nascent cartilage between WT and *Sec23a*$^{gt/gt}$ embryos at E11.5.

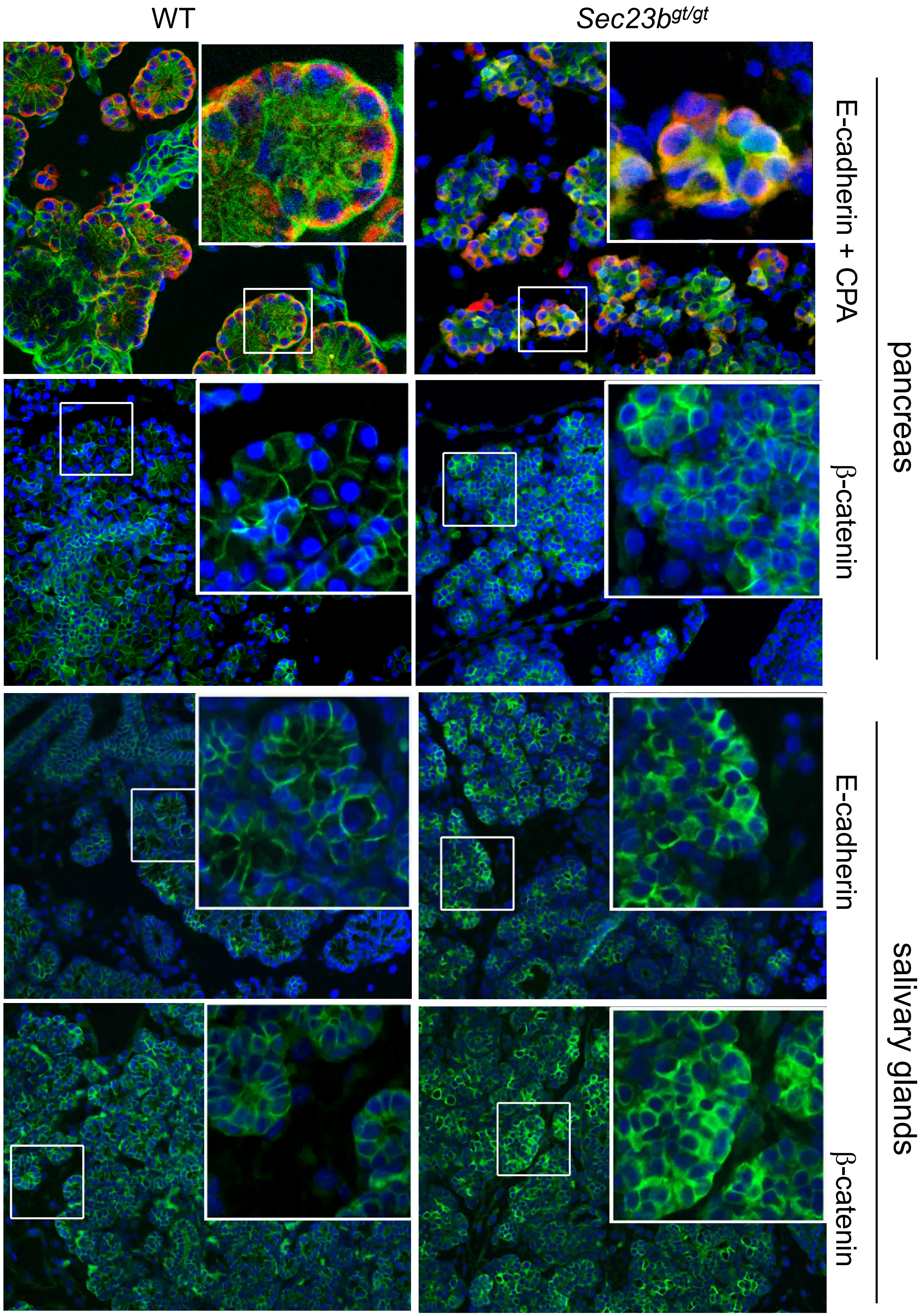
WT
Sec23b^gt/gt
E-cadherin + CPA
β-catenin
pancreas
E-cadherin
β-catenin
salivary glands

Figure S5. E-cadherin and β-catenin are mislocalized in *Sec23b*$^{gt/gt}$ pancreas and salivary glands. Shown are E-cadherin (green), CPA (red), β-catenin (green), and Dapi (blue) staining patterns in E15.5 pancreas and E15.5 salivary glands.
